# Multiphasic CT-Based Radiomics Analysis for the Differentiation of Benign and Malignant Parotid Tumors

**DOI:** 10.3389/fonc.2022.913898

**Published:** 2022-06-30

**Authors:** Qiang Yu, Anran Wang, Jinming Gu, Quanjiang Li, Youquan Ning, Juan Peng, Fajin Lv, Xiaodi Zhang

**Affiliations:** ^1^Department of Radiology, The First Affiliated Hospital of Chongqing Medical University, Chongqing, China; ^2^Philips Healthcare, Chengdu, China

**Keywords:** radiomics, machine learning, multiphasic CT, parotid tumors, differentiation

## Abstract

**Objective:**

This study aims to investigate the value of machine learning models based on clinical-radiological features and multiphasic CT radiomics features in the differentiation of benign parotid tumors (BPTs) and malignant parotid tumors (MPTs).

**Methods:**

This retrospective study included 312 patients (205 cases of BPTs and 107 cases of MPTs) who underwent multiphasic enhanced CT examinations, which were randomly divided into training (*N* = 218) and test (*N* = 94) sets. The radiomics features were extracted from the plain, arterial, and venous phases. The synthetic minority oversampling technique was used to balance minority class samples in the training set. Feature selection methods were done using the least absolute shrinkage and selection operator (LASSO), mutual information (MI), and recursive feature extraction (RFE). Two machine learning classifiers, support vector machine (SVM), and logistic regression (LR), were then combined in pairs with three feature selection methods to build different radiomics models. Meanwhile, the prediction performances of different radiomics models based on single phase (plain, arterial, and venous phase) and multiphase (three-phase combination) were compared to determine which model construction method and phase were more discriminative. In addition, clinical models based on clinical-radiological features and combined models integrating radiomics features and clinical-radiological features were established. The prediction performances of the different models were evaluated by the area under the receiver operating characteristic (ROC) curve (AUC) and the drawing of calibration curves.

**Results:**

Among the 24 established radiomics models composed of four different phases, three feature selection methods, and two machine learning classifiers, the LASSO-SVM model based on a three-phase combination had the optimal prediction performance with AUC (0.936 [95% CI = 0.866, 0.976]), sensitivity (0.78), specificity (0.90), and accuracy (0.86) in the test set, and its prediction performance was significantly better than with the clinical model based on LR (AUC = 0.781, *p* = 0.012). In the test set, the combined model based on LR had a lower AUC than the optimal radiomics model (AUC = 0.933 vs. 0.936), but no statistically significant difference (*p* = 0.888).

**Conclusion:**

Multiphasic CT-based radiomics analysis showed a machine learning model based on clinical-radiological features and radiomics features has the potential to provide a valuable tool for discriminating benign from malignant parotid tumors.

## Introduction

Salivary gland tumors are relatively rare and most commonly occur in parotid glands, with benign tumors accounting for about 75% (1, 2). Surgery is the primary treatment for parotid tumors, but the clinical choices of surgical methods for benign parotid tumors (BPTs) and malignant parotid tumors (MPTs) are quite different. Local or superficial parotidectomy is the main treatment for BPTs, while more aggressive approaches are used in MPTs, including total or subtotal parotidectomy, even facial nerve resection, or postoperative chemoradiation (2–4). Therefore, accurate preoperative identification is critical to the choice of treatment and prognosis for patients. At present, ultrasound-guided core biopsy (CB) and fine-needle aspiration (FNA) are the major methods for preoperative differentiation of the types of parotid tumors, with the risk of serious surgical complications, such as facial paralysis and tumor implantation metastasis (5, 6). Additionally, ultrasound imaging of deep-lobe parotid tumors is occluded by tissue structures such as the mandible, which affects the evaluation of the tumors, thus making sampling difficult and the accuracy of the results largely dependendent on the operator’ experience (2). Image examination is an important link in the achievement of accurate preoperative diagnosis of parotid tumors. For tumors occurring in the superficial lobe of parotid glands, ultrasound is the preferred method of examination, with limited value in the diagnosis of parotid tumors (7). CT and MRI are widely applied in preoperative localization, tumor invasion diagnoses, and differential diagnoses of parotid tumors, but conventional image evaluation largely depends on semantic features, and a large amount of information on tumor heterogeneity cannot be quantitatively elucidated (8). Although the application of multiparametric MRI in parotid tumors has increased, such as diffusion-weighted imaging (DWI) and dynamic contrast-enhanced MRI, its value in the differential diagnosis of benign and malignant parotid tumors is still controversial (9, 10).

In recent years, the application of radiomics to tumor diagnosis and treatment has been extensively studied. With the characteristics of high-throughput extraction of quantitative data from medical images in a noninvasive manner to explain the tumor heterogeneity, radiomics has rapidly developed into an emerging field in precision medicine (11). A previous study on the application of conventional CT radiomics to the differentiation of lympho-associated benign and malignant lesions of the parotid gland showed that it has a high differential ability (12). Another study demonstrated that dual-energy CT-based radiomics has a potential value in the differentiation of Warthin tumors from pleomorphic adenoma (13). However, this study only discussed the differential diagnosis of benign parotid tumors, and the sample size was relatively small. Xu et al. constructed a machine learning model based on the radiomics features extracted from plain and arterial phase scanning CT images to distinguish BPTs from MPTs (14), but only one machine learning classifier was utilized in their study. To the best of our knowledge, no studies have reported which phases, feature selection methods, and classifiers or their possible combinations are more discriminating in BPTs and MPTs. This will help in guiding the selection of the best model and phase for future multicenter studies of large datasets. This study aims to establish and validate machine learning prediction models based on CT radiomics features, clinical-radiological features, and a combination of the two types of features, and investigate their value in differentiating benign and malignant parotid tumors. At the same time, the prediction ability of differently combined radiomics models for BPTs and MPTs in the single-phase and multiphase were compared.

## Materials and Methods

### Patient Cohorts

This retrospective study was approved by the institutional review board of our hospital (approval ID: 2020080), and the requirement for obtaining written informed consent from patients was waived.

**Figure 1:**
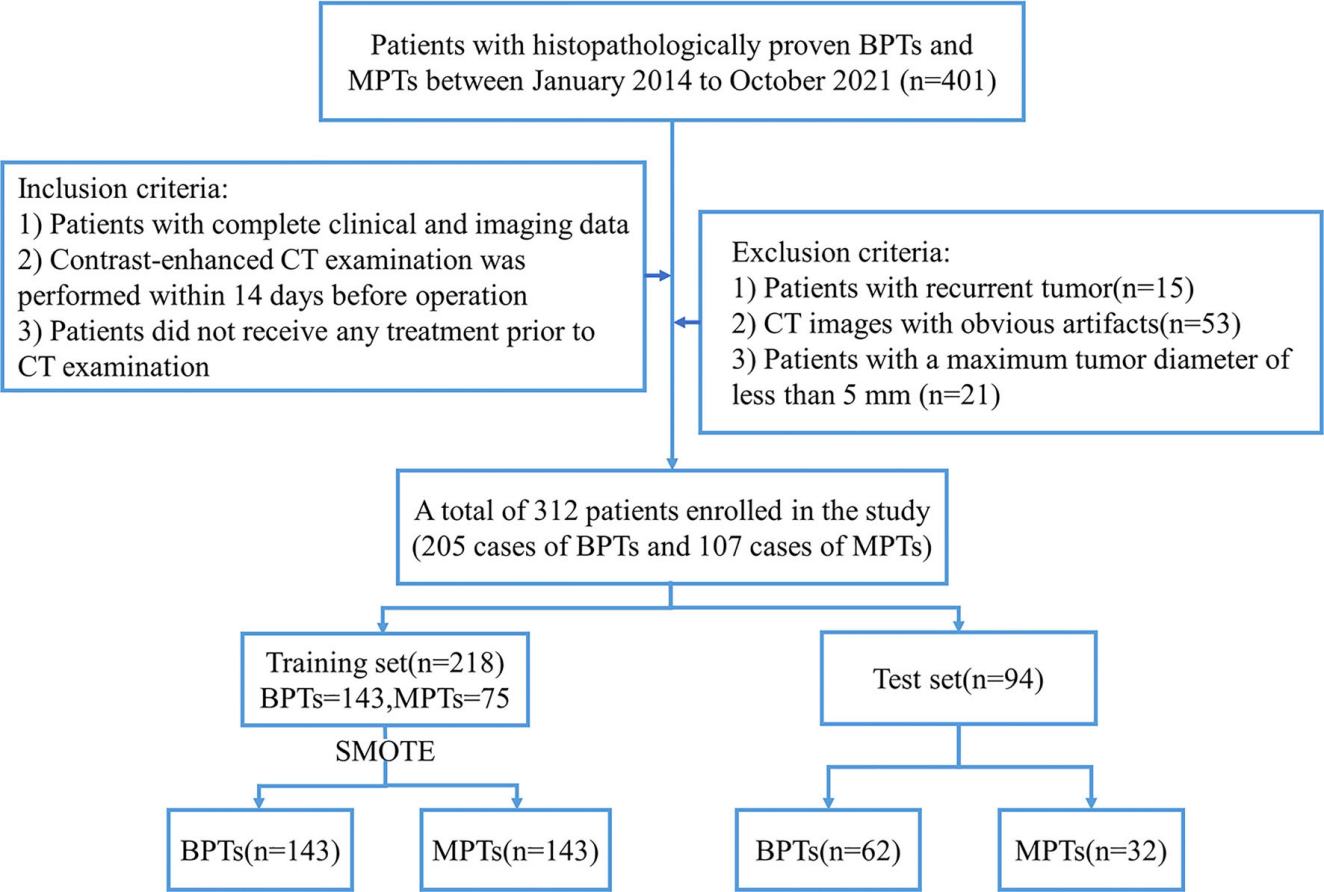
Flowchart of patient recruitment. BPTs, benign parotid tumors; MPTs, malignant parotid tumors; SMOTE, the synthetic minority oversampling technique.

Multiphasic enhanced CT images of patients with pathologically confirmed BPTs and MPTs from January 2014 to October 2021 were collected through a picture archiving and communication system (PACS). The inclusion criteria were as follows (1): patients with complete clinical and imaging data (2); contrast-enhanced CT examination was performed within 14 days before operation; and (3) patients did not receive any treatment prior to CT examination. The exclusion criteria were as follows (1): patients with recurrent tumor (2); CT images with obvious artifacts (3); patients with a maximum tumor diameter of less than 5 mm. Finally, 312 patients with parotid tumors were included, including 205 cases of BPTs and 107 cases of MPTs. The flowchart of patient recruitment is presented in **Figure 1**. **Supplementary Table S1** provides details of the pathological types of all patients with parotid tumors.

### CT Image Acquisition

Axial three-phase scanning (including plain scan, arterial phase, and venous phase) was performed on each patient by a multislice spiral CT scanner. The CT scanners were as follows (1): Discovery CT750 HD (GE Healthcare, Milwaukee, WI, USA) (2), SOMATOM Definition Flash (Siemens Healthcare, Forchheim, Germany), and (3) SOMATOM Definition Force (Siemens Healthcare, Forchheim, Germany). The scan was performed from the skull base to the thoracic entrance. After the completion of plain scanning, the contrast agent iohexol (300 mg/ml) was injected at a flow rate of 3~4 ml/s, followed by 30 ml of normal saline with the dosage of contrast agent (1.5 ml/kg). Arterial and venous phase images were acquired 35 and 60 s after the contrast injection, respectively. The acquisition parameters of the above different devices are introduced in detail in **Supplementary Table S2**.

### Clinical-Radiological Feature Evaluation

Univariate analysis was used to determine the statistically significant clinical-radiological features used for clinical model establishment. Clinical factors were collected, including, gender, age, and smoking history. The qualitative analysis of CT radiological features was evaluated by radiologists with 5 and 10 years of working experience (radiologists A and B, respectively), without knowing the pathological results of tumors. In the case of inconsistent evaluation results, the final results were obtained by a consensus between the two readers. The evaluation included the following radiological features (1): size (maximum tumor diameter in axial position) (2); tumor location (superficial or deep lobe was determined according to the main part of the tumor; the superficial and deep lobes are demarcated by a virtual line drawn from the lateral border of the posterior belly of the digastric muscle and retromandibular vein to the lateral edge of the mandible (15) (3); scope (localized or diffused; tumor involving local or whole parotid gland) (4); number (single or multiple) (5); shape (regular or irregular) (6); tumor margin (well-defined or ill-defined) (7); cystic or necrotic areas (CNA; absent or present) (8); infiltration of surrounding tissue (IST, absent or present; tumors involve surrounding muscles, bone, skin, or subcutaneous tissue (3) (9); lymphatic metastasis (LM; absent or present; obvious density change in cervical lymph nodes or short axis diameter >10 mm) (10); CT value at each phase (the solid section of the tumor was measured three times, and the average was calculated) (11); enhancement degree in P-A phase (difference in CT values between arterial and plain phases); and (12) enhancement degree in A-V phase (difference in CT values between venous and arterial phases).

### Radiomics Feature Extraction and Selection

The CT images of all patients were loaded into the open-source image processing platform ITK-SNAP software (version 3.6.0, http://www.itksnap.org) in DICOM format. The 3D volume of interest (VOI) of the tumor was formed by manual delineation layer by layer along the contour of the tumor on the plain scan, arterial phase, and venous phase CT images by radiologist A. In order to evaluate the reproducibility of features, 30 cases of CT images from each phase were randomly selected to evaluate intra- and interobserver agreement of radiomics features. Radiologist A performed the second tumor VOI segmentation at intervals of 1 week after the first tumor VOI segmentation, and radiologist B performed the tumor VOI segmentation independently. The intraclass correlation coefficient (ICC) was used to evaluate the intra- and interobserver agreement, and ICC values greater than 0.75 indicated good agreement.

PyRadiomics version 3.0 was used for feature extraction and image preprocessing. In order to reduce the influence of different scanning devices, the images were resampled to a voxel spacing of 1 × 1 × 1 mm³. The gray value discretization of the image was performed by the use of a fixed bin width of 25 HU to normalize image intensity and reduce image noise. In total, 851 features were extracted from each VOI, including the following four types of features (1): first-order statistics (18 features) (2), shape-based (14 features) (3), texture classes (75 features), and (4) wavelet features (744 features). The features of CT phase extraction at each phase were recorded in **Supplementary Excel S1**. The calculation formula and definition of the above features were provided in PyRadiomics documentation (https://pyradiomics.readthedocs.io/en/latest).

**Figure 2:**
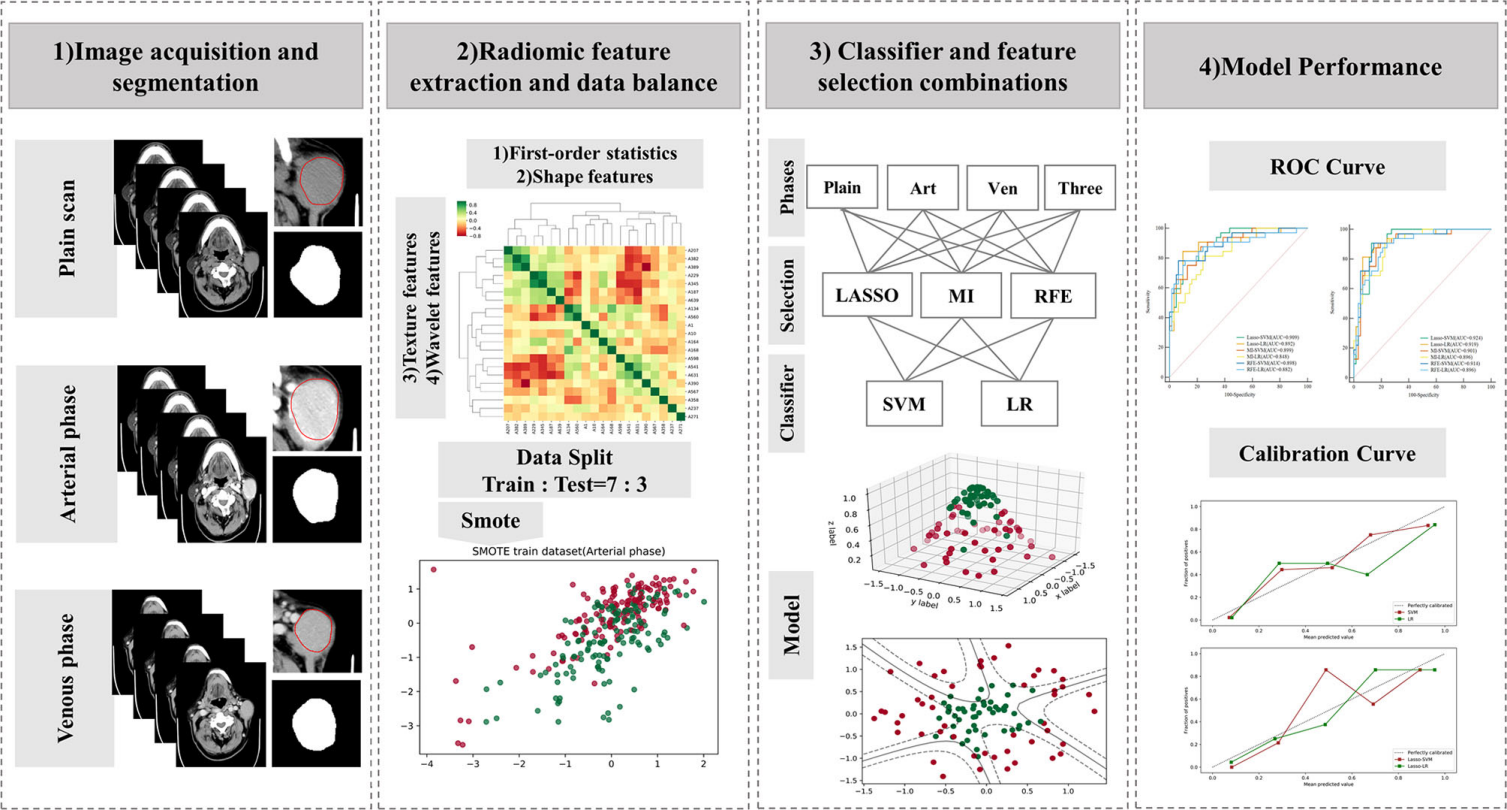
Workflow of the radiomics analysis. LASSO, least absolute shrinkage and selection operator; MI, mutual information; RFE, recursive feature elimination; SVM, support vector machine; LR, logistic regression.

Before feature screening, all patients were randomized 7:3 into training and test sets. Feature selection plays an important role in reducing the task difficulty of the model and preventing model overfitting. The Mann–Whitney *U* test (*p* < 0.05) was used to initially screen the radiomics features (*ICC >*0.75). Before further feature screening, all features were normalized by the use of a Z-score to reduce the influence of different dimensions among features. In addition, in order to alleviate the impact of sample imbalance, the synthetic minority oversampling technique (SMOTE) was adopted to balance minority samples in the training set, thus leading to a sample proportion of 1:1. The algorithm has been proven to be helpful in avoiding overfitting of the model in the unbalanced data set and improve the overall generalization ability (16–18). On the balanced data set, least absolute shrinkage and selection operator (LASSO), mutual information (MI), and recursive feature extraction (RFE) were applied for radiomics feature further screening. **Figure 2** shows the workflow of radiomics analysis.

### Radiomics Model Establishment and Validation

The clinical model served as the baseline model for the comparison between different models in our study. Radiomics models based on the CT radiomics features in each phase and combined models were also established. The combined model was established by the integration of the clinical-radiological features and radiomics features screened by LASSO in the arterial phase. There are significant differences in the prediction performance of models among different combinations of various machine learning classifiers and feature selection methods (19–21). Based on the radiomics features extracted from the plain scan, arterial, venous, and combined phase CT images, two common machine learning classifiers, support vector machine (SVM) and logistic regression (LR), were combined in pairs with three feature selection methods (LASSO, MI, and RFE) to generate a total of 24 models to determine the best performing radiomics model. In the training set, GridSearchCV (CV = 5, namely, 5-fold cross-validation) was used to optimize the hyperparameters of the model to reduce its training error and generalization one. For each model, the prediction performance of the machine learning model was evaluated by sensitivity, specificity, accuracy, and the area under the receiver operating characteristic (ROC) curve (AUC). In addition, the calibration curve was plotted to assess the calibration of models in the test set.

### Statistical Analysis

Python version 3.7.3 and R version 3.6.0 were used to complete model establishment and statistical analysis. Quantitative variables were expressed as mean ± standard deviation (SD) or median and interquartile range (IQR), and categorical variables were expressed as numbers. An independent samples *t*-test or Mann–Whitney *U* test was adopted for quantitative variables, and the chi-square test or Fisher’s exact test was used for categorical variables. The comparison of AUC differences between different models was completed by the Delong test. The level of significance was set at *p* < 0.05. The “imbalanced learn version 0.8.1” package was applied for data balancing in the training set. The feature screening and machine learning classifier construction were performed by the “scikit learn version 1.0.1” package.

## Results

### Clinical-Radiological Factors and Clinical Models

**Figure 3:**
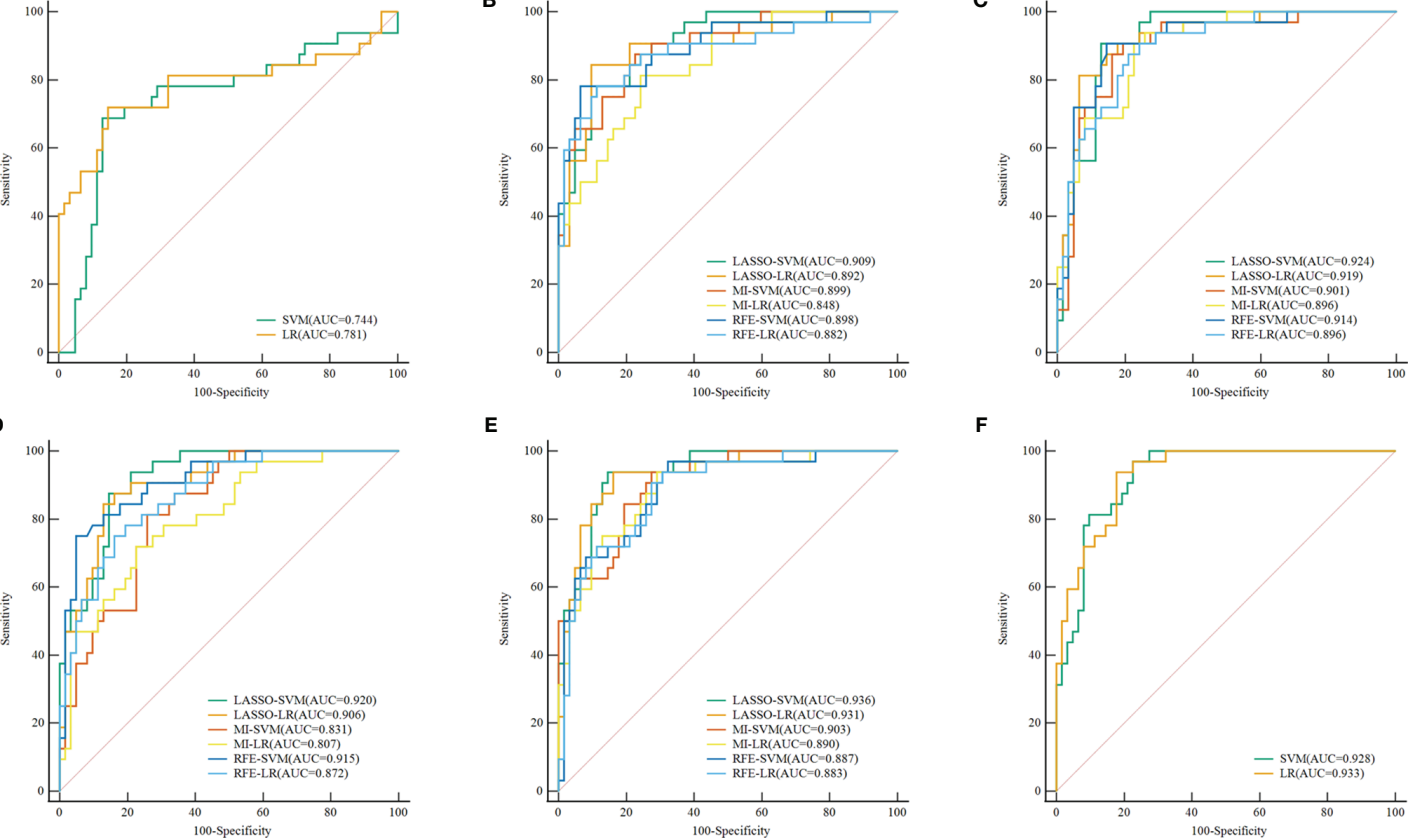
ROC curve of the clinical, radiomics, and combined models in the test set, respectively. **(A)** ROC curve of Clinical models. **(B–E)** ROC curve of radiomics models (different combinations of 3 feature selection methods and 2 classifiers) based on the plain scan, arterial phase, venous phase, and three-phase combination. **(F)** ROC curve of combined models. ROC, receiver operating characteristic; LASSO, least absolute shrinkage and selection operator; MI, mutual information; RFE, recursive feature elimination; SVM, support vector machine; LR, logistic regression.

The baseline table of clinical-radiological features of patients is shown in **Table 1**. In the training and test sets, there were significant differences in size, scope, shape, margin, IST, and LM between BPT and MPT groups (*p* < 0.05). Although the tumor location was not significantly different in the training set, a meta-analysis showed that tumor location (superficial lobe or deep lobe) may be a useful marker to help distinguish BPTs and MPTs (22). Therefore, the abovementioned seven clinical-radiological features were applied to the establishment of clinical models. In the established clinical models based on SVM and LR, the overall efficiency of the LR-based model was higher than that of the SVM-based one. The prediction performance of the LR-based model: in the training set, the AUC was 0.769 [95% CI = 0.716, 0.817], sensitivity was 0.50, specificity was 0.91, and accuracy was 0.71; in the test set, the AUC was 0.781 [95% CI = 0.684, 0.860], sensitivity was 0.53, specificity was 0.89, and accuracy was 0.77; the details of clinical models are shown in **Table 2**. The results showed that the prediction ability of clinical models for malignant parotid tumors was relatively low. **Figure 3A** shows the ROC curve of the clinical model in the test set.

**Table 1:** Clinical-radiological features of the training and test sets.

Clinical-radiological features	Training set (*n* = 218)			Test set (*n* = 94)		
	BPTs (*n* = 143)	MPTs (*n* = 75)	*p*-value	BPTs (*n* = 62)	MPTs (*n* = 32)	*p*-value
Gender^a^ (M/F)	75/68	39/36	0.950	37/25	17/15	0.543
Age^b^ (year)	55.00 (15.00)	48.00 (24.50)	0.023	53.69 (14.34)	51.38 (16.54)	0.483
Smoke^a^ (absent/present)	81/62	47/28	0.391	34/28	21/11	0.315
Size^b^ (mm)	19.77 (8.43)	23.09 (12.01)	0.003	20.84 (8.46)	28.83 (14.70)	0.001
Location^a^ (superficial/deep lobe)	111/32	50/25	0.080	57/5	16/16	<0.001
Scope^a^ (localized/diffuse)	143/0	65/10	<0.001	62/0	23/9	<0.001
Number^a^ (single/multiple)	136/7	73/2	0.669	59/3	31/1	1.000
Shape^a^ (regular/irregular)	96/47	36/39	0.006	51/11	14/18	<0.001
Margin^a^ (well-defined/ill-defined)	123/20	44/31	<0.001	57/5	17/15	<0.001
CNA^a^ (absent/present)	77/66	39/36	0.795	42/20	23/9	0.681
IST^a^ (absent/present)	137/6	44/31	<0.001	57/5	17/15	<0.001
LM^a^ (absent/present)	143/0	68/7	0.001	62/0	28/4	0.021
**CT value**^**b**^ **(HU)**						
Plain	42.00 (15.00)	45.00 (16.50)	0.524	43.15 (11.94)	43.97 (13.26)	0.761
Arterial	84.00 (41.00)	79.00 (38.50)	0.591	86.69 (36.32)	84.31 (26.36)	0.743
Venous	86.00 (29.00)	87.00 (31.50)	0.961	86.94 (23.71)	89.50 (26.85)	0.636
Art-Pl	38.00 (36.50)	34.00 (30.00)	0.209	34.00 (39.50)	33.00 (24.75)	0.886
Ven-Art	3.07 (23.72)	5.03 (18.21)	0.499	6.00 (28.75)	8.00 (16.75)	0.330

a Categorical data as numbers (n).

b Quantitative data are mean (standard deviation) or median (quartile). p-value was calculated with independent samples t-test or Mann–Whitney U test. p-value was calculated with the χ^2^ or Fisher’s exact test. BPTs, benign parotid tumors; MPTs, malignant tumors; F, female; M, male; CNA, cystic or necrotic areas; LM, lymphatic metastasis; CT, computed tomography; Art, arterial phase; Pl, plain scan; Ven, venous phase.

**Table 2:** Diagnostic performance of the clinical, radiomics, and combined models.

Model	AUC (95%CI)	Sensitivity	Specificity	Accuracy	*p*-value
**Training set**					
Clinical model (LR)	0.769 (0.716 to 0.817)	0.50	0.91	0.71	–
Clinical model (SVM)	0.871 (0.827 to 0.908)	0.66	0.93	0.80	<0.001
Radiomics model 1	0.951 (0.919 to 0.973)	0.84	0.92	0.88	<0.001
Radiomics model 2	0.991 (0.972 to 0.999)	0.96	0.97	0.96	<0.001
Combined model (LR)	0.950 (0.918 to 0.972)	0.85	0.94	0.90	<0.001
Combined model (SVM)	0.978 (0.854 to 0.992)	0.92	0.97	0.95	<0.001
**Test set**					
Clinical model (LR)	0.781 (0.684 to 0.860)	0.53	0.89	0.77	–
Clinical model (SVM)	0.744 (0.644 to 0.828)	0.69	0.85	0.80	0.539
Radiomics model 1	0.924 (0.850 to 0.968)	0.78	0.89	0.85	0.012
Radiomics model 2	0.936 (0.866 to 0.976)	0.78	0.90	0.86	0.013
Combined model (LR)	0.933 (0.862 to 0.974)	0.75	0.85	0.82	0.006
Combined model (SVM)	0.928 (0.856 to 0.971)	0.81	0.87	0.85	0.009

The p-value was calculated by the Delong test. AUC, area under the receiver operating characteristic curve; CI, confidence interval; SVM, support vector machine; LR, logistic regression. Radiomics model 1, the best radiomics model based on a single phase (arterial-LASSO-SVM). Radiomics model 2, the best radiomics model based on the multiphasic phase (three-LASSO-SVM).

### Radiomics Feature Selection and Radiomics Models

Among the 851 radiomics features extracted from each phase, there were 680 highly repeatable features in the plain scan, 651 in the arterial phase, and 667 in the venous phase, respectively (ICC >0.75). The ICC analysis results are shown in **Supplementary Excel S2**. After preliminary screening by the Mann–Whitney *U* test, SMOTE was adopted to balance the minority class for samples in the training set, with the proportion of samples in the training set adjusted to 1:1 (BPTs = 143, MPTs = 143). Three feature screening methods (LASSO, MI, and RFE) combined with two machine learning methods (SVM and LR) were then used to establish 24 radiomics models in the plain scan, arterial phase, and venous phase, as well as a three-phase combination. The results of different feature screening methods in each phase are shown in **Supplementary Table S3**.

**Figure 4:**
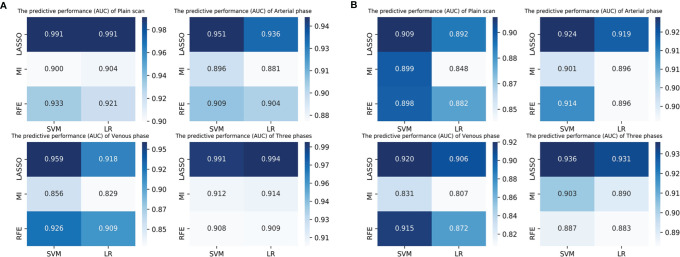
The predictive performance (AUC) of radiomics models (3 feature selection methods and 2 classifiers) based on different phases in the training set **(A)** and test set **(B)**. LASSO, least absolute shrinkage and selection operator; MI, mutual information; RFE, recursive feature elimination; SVM, support vector machine; LR, logistic regression.

The discrimination indicators of all radiomics models are shown in **Table 3**. **Figures 3B–E** show the ROC curve of all radiomics models in the test set. The AUC values of all radiomics models ranged from 0.819 to 0.994 in the training set and 0.807 to 0.936 in the test set (**Figure 4**). Among all radiomics models, LASSO-SVM models based on three-phase combination had the highest discrimination efficiency: in the training set, the AUC was 0.991 [95% CI = 0.972, 0.999], sensitivity was 0.96, specificity was 0.97, and accuracy was 0.96; in the test set, the AUC was 0.936 [95% CI = 0.866, 0.976], sensitivity was 0.78, specificity was 0.90, and accuracy was 0.86; in the test set, the prediction performance was significantly better than that of the LR-based clinical models (*p* = 0.012, Delong test). Among the three feature screening methods, the efficiency of LASSO combined with SVM or LR machine learning classifier was generally better than that of MI and RFE feature screening methods.

**Table 3:** Diagnostic performance of the radiomics models.

Model	SVM			LR	
	Training	Test		Training	Test
**Arterial phase**					
LASSO					
AUC (95% CI)	0.951 (0.919 to 0.973)	0.924 (0.850 to 0.968)		0.936 (0.901 to 0.961)	0.919 (0.845 to 0.966)
Sensitivity	0.84	0.78		0.86	0.81
Specificity	0.92	0.89		0.90	0.94
Accuracy	0.88	0.85		0.88	0.89
ML					
AUC (95% CI)	0.896 (0.855 to 0.929)	0.901 (0.822 to 0.953)		0.881 (0.838 to 0.916)	0.896 (0.816 to 0.950)
Sensitivity	0.78	0.75		0.81	0.78
Specificity	0.88	0.85		0.81	0.79
Accuracy	0.83	0.82		0.81	0.79
RFE					
AUC (95% CI)	0.909 (0.869 to 0.940)	0.914 (0.838 to 0.962)		0.904 (0.863 to 0.935)	0.896 (0.816 to 0.950)
Sensitivity	0.81	0.84		0.80	0.72
Specificity	0.90	0.87		0.87	0.84
Accuracy	0.86	0.86		0.84	0.80
**Venous phase**					
LASSO					
AUC (95% CI)	0.959 (0.929 to 0.979)	0.920 (0.846 to 0.966)		0.918 (0.880 to 0.947)	0.906 (0.828 to 0.957)
Sensitivity	0.89	0.69		0.82	0.78
Specificity	0.87	0.87		0.82	0.87
Accuracy	0.88	0.81		0.82	0.84
ML					
AUC (95% CI)	0.856 (0.810 to 0.894)	0.831 (0.739 to 0.900)		0.829 (0.780 to 0.871)	0.807 (0.713 to 0.882)
Sensitivity	0.75	0.81		0.73	0.75
Specificity	0.78	0.74		0.74	0.73
Accuracy	0.77	0.77		0.73	0.73
RFE					
AUC (95% CI)	0.926 (0.889 to 0.954)	0.915 (0.839 to 0.963)		0.909 (0.869 to 0.940)	0.872 (0.787 to 0.932)
Sensitivity	0.80	0.72		0.81	0.69
Specificity	0.89	0.95		0.83	0.87
Accuracy	0.85	0.87		0.82	0.81
**Plain scan**					
LASSO					
AUC (95% CI)	0.991 (0.972 to 0.998)	0.909 (0.832 to 0.959)		0.991 (0.972 to 0.998)	0.892 (0.811 to 0.947)
Sensitivity	0.94	0.72		0.96	0.81
Specificity	0.95	0.90		0.94	0.90
Accuracy	0.94	0.84		0.95	0.87
ML					
AUC (95% CI)	0.900 (0.859 to 0.932)	0.899 (0.819 to 0.951)		0.904 (0.864 to 0.935)	0.848 (0.759 to 0.914)
Sensitivity	0.77	0.75		0.84	0.62
Specificity	0.86	0.87		0.85	0.84
Accuracy	0.81	0.83		0.84	0.77
RFE					
AUC (95% CI)	0.933 (0.897 to 0.959)	0.898 (0.819 to 0.951)		0.921 (0.884 to 0.950)	0.882 (0.799 to 0.939)
Sensitivity	0.81	0.78		0.85	0.78
Specificity	0.90	0.92		0.84	0.87
Accuracy	0.85	0.87		0.84	0.84
**Three-phase combination**					
LASSO					
AUC (95% CI)	0.991 (0.972 to 0.999)	0.936 (0.866 to 0.976)		0.994 (0.977 to 1.000)	0.931 (0.859 to 0.973)
Sensitivity	0.96	0.78		0.97	0.75
Specificity	0.97	0.90		0.97	0.94
Accuracy	0.96	0.86		0.97	0.87
ML					
AUC (95% CI)	0.912 (0.873 to 0.942)	0.903 (0.824 to 0.954)		0.914 (0.875 to 0.943)	0.890 (0.809 to 0.945)
Sensitivity	0.81	0.69		0.83	0.75
Specificity	0.86	0.84		0.86	0.84
Accuracy	0.84	0.79		0.85	0.81
RFE					
AUC (95% CI)	0.908 (0.869 to 0.939)	0.887 (0.805 to 0.943)		0.909 (0.870 to 0.940)	0.883 (0.800 to 0.940)
Sensitivity	0.81	0.69		0.82	0.72
Specificity	0.89	0.85		0.85	0.89
Accuracy	0.85	0.80		0.84	0.83

AUC, area under the receiver operating characteristic curve; CI, confidence interval; SVM, support vector machine; LR, logistic regression; LASSO, least absolute shrinkage and selection operator; MI, mutual information; RFE, recursive feature elimination.

### The Performance of Phases and Combined Model

For single phase, the performance of models based on the arterial phase was generally better than that in the venous and plain scan phases. LASSO-SVM model based on the arterial phase has the highest prediction performance: in the test set, the AUC was 0.924 [95% CI = 0.850, 0.968], sensitivity was 0.78, specificity was 0.89, and accuracy was 0.85. It is worth noting that although the prediction performance of the radiomics models based on the plain scan was generally lower than that of those based on arterial and venous phases, it also achieved a high one. The AUC of LASSO-SVM models based on plain scan in the test set was 0.909 [95% CI = 0.832, 0.959], which was significantly higher than that of LR-based clinical model (AUC = 0.781, *p* = 0.045, Delong test). For multiphase, the LASSO-SVM model based on a three-phase combination achieved the best prediction performance in all phases, which was constructed with 62 radiomics features obtained from multiphase sequences (three-phase combination) by LASSO. However, too many features will increase the complexity of the model. Therefore, in order to avoid overfitting caused by more features, we chose to integrate 21 radiological features from the single phase (arterial phase) by LASSO with clinical-radiological features to establish combined models.

**Figure 5:**
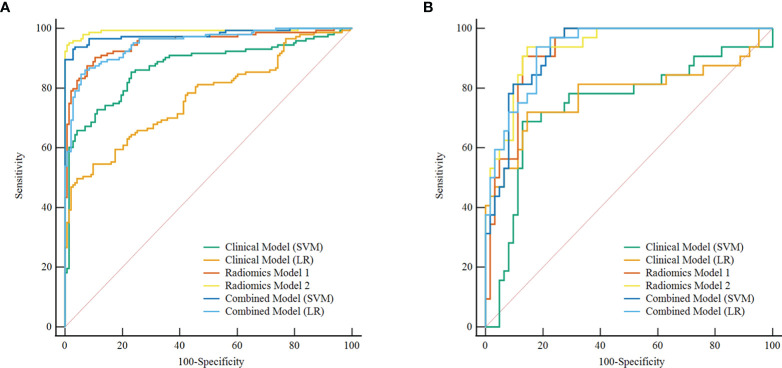
Comparison of ROC curve between different models in the training set **(A)** and test set **(B)**. ROC, receiver operating characteristic; SVM, support vector machine; LR, logistic regression. Radiomics model 1, the best radiomics model based on a single phase (arterial-LASSO-SVM); Radiomics model 2, the best radiomics model based on a multiphasic phase (three-LASSO-SVM).

**Figure 6:**
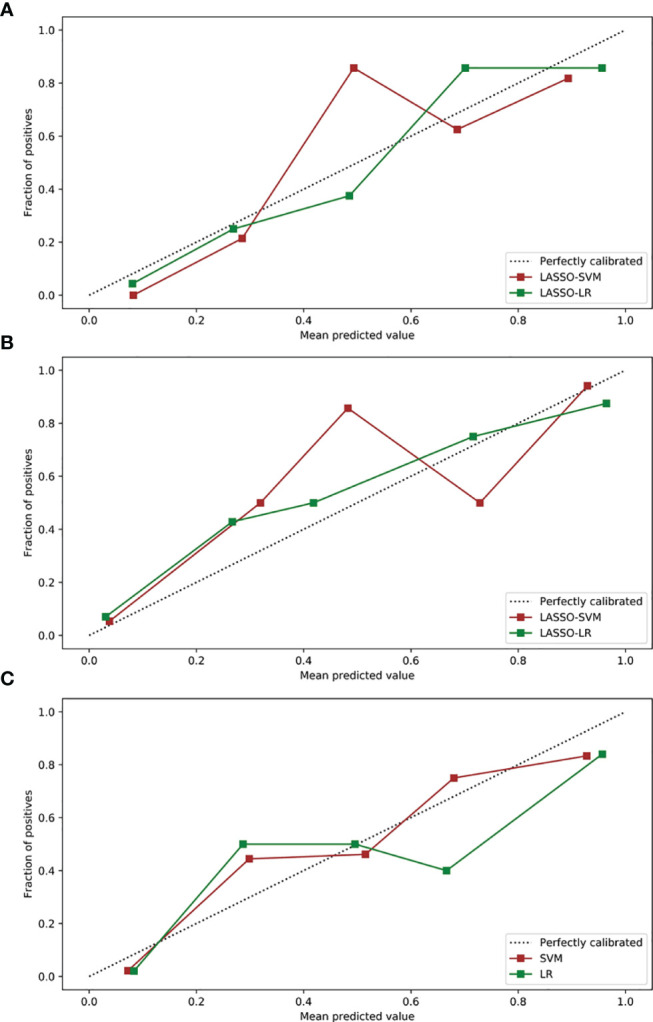
Calibration curves of radiomics and combined models in the test sets. **(A)** Calibration curves of radiomics models based on arterial phase. **(B)** Calibration curves of radiomics models based on a three-phase combination. **(C)** Calibration curves of combined models. LASSO, least absolute shrinkage, and selection operator; SVM, support vector machine; LR, logistic regression.

In the training and test sets, the prediction performance of the combined model was better than that of the clinical model. The prediction performance of LR-based combined model in the test set was as follows: the AUC was 0.933 [95% CI = 0.862, 0.974], sensitivity was 0.75, specificity was 0.85, and accuracy was 0.82. The diagnostic efficiency of the LR-based combined model was significantly better than that of the LR-based clinical model (AUC = 0.933 vs. 0.781, *p* = 0.006, Delong test); however, the prediction performance of the LR-based combined mode (AUC = 0.933 vs. 0.936, *p* = 0.888, Delong test) was similar to that of the three-phase combined LASSO-SVM radiomics model (**Table 2**). **Figure 3F** depicts the ROC curve of combined models in the test set. A comparison of the ROC curve between different models (clinical models, the optimal radiomics models base on single-phase and multiphase, and combined modes) in the training set (**A**) and test set (**B**) is shown in **Figure 5**. The calibration curves of the LASSO radiomics model based on the arterial phase, the LASSO radiomics model based on a three-phase combination, and the combined models are shown in **Figure 6**. These models all showed good calibration performance.

## Discussion

In this study, prediction models of benign and malignant parotid tumors based on clinical-radiological features, radiomics features, and combined features were established and validated. Among the 28 established prediction models, radiomics models and combined ones achieved outstanding performance. More specifically, in the independent test set, the LASSO-SVM radiomics model (AUC = 0.936) based on a three-phase combination and the LR-based combined model (AUC = 0.933) had higher prediction accuracy in the differentiation of BPTs from MPTs compared with the optimal clinical model based on LR (AUC = 0.781, *p* = 0.012, *p* = 0.006), and they showed better calibration ability. This suggested that the developed models may be helpful to the preoperative diagnosis of BPTs and MPTs.

In conventional image diagnosis, diffuse tumor growth patterns, irregular shape, ill-defined margin, deep lobe lesions, surrounding tissue involvement, and lymphatic metastasis are considered to be more common in MPTs (22–24). The results of this study are consistent with those of previous studies. A meta-analysis showed that the sensitivities of US, CT, and MRI in the differentiation of benign and malignant tumors of salivary glands were 0.66, 0.70, and 0.80, respectively (25). Another conventional MRI analysis showed that the sensitivities of the diagnosis of MPTs only by infiltration of surrounding tissue and irregular shape were 0.68 and 0.16, respectively (23). These showed that the overlapping imaging features between BPTs and MPTs are the main limitations of conventional radiology diagnoses. In this study, the AUC values of SVM and LR clinical models based on clinical-radiological features (size, scope, shape, margin, location, IST, and LM) were 0.744 and 0.781 respectively, in the test set, but the sensitivities to the two models were only 0.69 and 0.53, respectively, which suggested that the prediction model established only by clinical-radiological features cannot differentiate BPTs from MPTs well. The radiomics features can reflect subtle differences between tumors that cannot be recognized by the naked eye. The diagnostic efficiency of established radiomics models (AUC = 0.807~0.936, sensitivity = 0.62~0.84) in the test set was better than that of the clinical models (AUC = 0.744, 0.781, sensitivity = 0.69, 0.53), and the overall prediction accuracy for malignant tumors significantly improved. For combined models, although the prediction performance of the optimal LR combined model (combined with 7 clinical factors and radiomics features screened by LASSO in the arterial phase) had no improvement on that of LASSO-SVM radiomics models based on a three-phase combination (AUC = 0.933 vs. 0.936), it had a great improvement on that of the LASSO-LR radiomics model based on the arterial phase (AUC = 0.933 vs. 0.919). This suggests that the mutual complementation of clinical-radiological features and radiomics ones has the greatest benefit for the diagnosis of BPTs and MPTs, which may benefit from important extratumoral features such as lymphatic metastasis and tumor infiltration into the surrounding tissue.

Among the screened radiomics features by three feature selection methods, the sphericity based on shape features was considered to be highly related to the diagnosis of BPTs and MPTs. Sphericity is a dimensionless metric that is independent of scale and orientation and may be applied to estimate the roundness of the shape of the tumor region relative to a circle; the closer the value is to 1, the closer the tumor is to the perfect sphere. A previous study showed that among the radiomics features extracted from T2WI images, the volume density AEE value related to sphericity was higher in Warthin tumors than in MPTs (26). In this study, the sphericity in the arterial phase was significantly different between BPTs and MPTs (*p* < 0.05, Mann–Whitney *U* test), with the value in BPTs generally higher. This quantitative index showed that the morphology of BPTs is more regular compared with MPTs. In addition, wavelet features had the highest weight in the radiomics labels screened by different feature screening methods, which indicates that wavelet features may reflect the spatial heterogeneity of tumors on several scales (27, 28).

In recent years, there have been many radiomics studies focused on the diagnosis of parotid tumors and the prediction of side effects related to radiotherapy (12–14, 29), but they are mainly based on MRI radiomics. Zheng et al. extracted the radiomics features of benign and malignant parotid tumors from TWI and T2WI sequences and established the radiomics nomogram model by multivariate logistic regression analysis (30). The AUC value of this model reached 0.938 in the differentiation of BPTs from MPTs, which was close to the prediction performance of our optimal model (AUC = 0.936). In addition, some scholars have also applied CT radiomics to the differentiation of benign and malignant lymph-related lesions of parotid glands and benign parotid tumors (12, 13), which have both shown excellent predictive performance. Xu et al. established an SVM-based combined prediction model based on the radiomics features extracted from plain CT scan and arterial phase combined with conventional CT image features to differentiate benign and malignant parotid tumors, and in the test set, model diagnosis results of BPTs and MPTs were: accuracy, 0.84; specificity, 0.74; and sensitivity, 0.82 (14). In contrast, the diagnostic efficiency of LASSO-SVM radiomics model based on three-phase combination (accuracy, 0.86; specificity, 0.90; sensitivity, 0.78) was relatively high. In contrast to the above research, in this study, the SMOTE algorithm was adopted to balance minority class samples in the training set and achieve a better class balance. At the same time, GridSearchCV was applied to the optimization of the model hyperparameters. All these methods effectively reduced the training error of models in the training set and improve overall prediction performance in the test set, which have also been validated in other studies (16–18). In addition, in this study, a variety of feature screening methods and machine learning classifiers were used. The results showed that the overall efficiency of the LASSO-SVM-based model was outstanding. Moreover, the prediction performances of different radiomics models were compared in different phases. The results showed that in the single phase, the prediction performance of the models based on the arterial phase was generally better than that of those based on plain scan and venous phase. It is worth mentioning that although the prediction performance of radiomics models based on the plain scan was lower than that of those based on the arterial phase, it was significantly better than that of clinical models. This research result may be beneficial to the popularization of the prediction model in patients with parotid tumors, especially for those who are not suitable for enhanced scanning.

Our study has limitations. First, this study was limited by the single-center studies and low incidence rate of parotid malignant tumors, with a small sample size of included parotid malignancies. Second, in this study, an independent internal test set was used to verify the reliability of the model without being supported by the external test set, and the generalization ability of models still needs to be further validated by multicenter prospective research. Third, deep learning has developed rapidly in the medical field, which has the ability to process image information more efficiently compared with the traditional machine learning classifier. In the future, deep learning could be applied to the multiclassification task of parotid tumors. Finally, the multiomics combination is the development trend of radiomics in the future, including radiogenomics, radiopathomics, and multiradiomics combinations based on different medical images. In future studies, the application of multiomics combined models to the diagnosis and treatment of parotid tumors can be explored.

## Conclusion

In conclusion, the radiomics models and the combined ones established in this study showed high prediction accuracy in the diagnosis of benign and malignant parotid tumors, with obvious advantages compared with conventional image diagnosis, which may provide a valuable tool for clinical decision-making of patients with parotid tumors.

## Data Availability Statement

The datasets presented in this study can be found in online repositories. The names of the repository/repositories and accession number(s) can be found in the article/**Supplementary Material**.

## Ethics Statement

The studies involving human participants were reviewed and approved by the Institutional Review Board of the First Affiliated Hospital of Chongqing Medical University. Written informed consent from the participants’ legal guardian/next of kin was not required to participate in this study in accordance with the national legislation and the institutional requirements. Written informed consent was obtained from the individual(s), and minor(s)’ legal guardian/next of kin, for the publication of any potentially identifiable images or data included in this article.

## Author Contributions

JP and QY designed the research. AW, JG, QL, and YN collected the data and preprocessed the data. QY, XZ, and JG performed major data analyses and drafted the manuscript. JP and FL participated in the review and editing. All authors contributed to the article and approved the submitted version.

## Funding

This project received support from The Foundation of Science and Technology Bureau of Yuzhong District, Chongqing, China (Grant No. 20190111) and the Natural Science Foundation of Chongqing, China (Grant No. cstc2021jcyj-msxmX0020).

## Conflict of Interest

Author XZ was employed by Philips Healthcare.

The remaining authors declare that the research was conducted in the absence of any commercial or financial relationships that could be construed as a potential conflict of interest.

## Publisher’s Note

All claims expressed in this article are solely those of the authors and do not necessarily represent those of their affiliated organizations, or those of the publisher, the editors and the reviewers. Any product that may be evaluated in this article, or claim that may be made by its manufacturer, is not guaranteed or endorsed by the publisher.
