# Supplementary material for: Multiphasic CT-Based Radiomics Analysis for the Differentiation of Benign and Malignant Parotid Tumors
Source: Front Oncol. 2022 Jun 30;12:913898. doi: 10.3389/fonc.2022.913898 (PMC9280642; doi:10.3389/fonc.2022.913898)
Supplement: Supplementary file 3 [file DataSheet_3.docx]

| **Table 1** Histopathological types of parotid tumors | | | | | | |
| --- | --- | --- | --- | --- | --- | --- |
| BPT |  | number |  | MPT |  | number |
| Pleomorphic adenoma |  | 93 |  | Mucoepidermoid carcinoma |  | 37 |
| Warthin tumor |  | 59 |  | Adenoid cystic carcinoma |  | 12 |
| Basal cell adenoma |  | 53 |  | Acinic cell carcinoma |  | 20 |
|  |  |  |  | Adenocarcinoma |  | 7 |
|  |  |  |  | Squamous cell carcinoma |  | 9 |
|  |  |  |  | Lymphoepithelial carcinoma |  | 2 |
|  |  |  |  | basal cell adenocarcinoma |  | 3 |
|  |  |  |  | Myoepithelial carcinoma |  | 6 |
|  |  |  |  | Salivary ductal carcinoma |  | 3 |
|  |  |  |  | Lymphoma |  | 6 |
|  |  |  |  | Carcinosarcoma |  | 2 |

Note: BPT, benign parotid tumors; MPT, malignant tumors

| **Table 2** CT Imaging Protocol | | | | | | |
| --- | --- | --- | --- | --- | --- | --- |
|  |  | Discovery  CT750 HD |  | SOMATOM  Definition Flash |  | SOMATOM  Definition Force |
| Tube voltage(kV) |  | 120 |  | 120 |  | 100 |
| Tube current(mAs) |  | 180 |  | 250 |  | 270 |
| Gantry rotation time(s) |  | 0.6 |  | 0.5 |  | 0.28 |
| Detector collimation(mm) |  | 64×0.625 |  | 128×0.6 |  | 128×0.6 |
| Slice thickness(mm) |  | 5 |  | 5 |  | 5 |
| Image matrix |  | 512×512 |  | 512×512 |  | 512×512 |

Note: CT computed tomography

| **Table 3 Radiomics feature selection results** | | | | |
| --- | --- | --- | --- | --- |
|  |  | Arterial phase |  | Venous phase |
| Lasso |  | original_shape_Elongation  original_shape_Sphericity  wavelet-LLH_glcm_MaximumProbability  wavelet-LLH_glrlm_RunVariance  wavelet-LLH_glszm_GrayLevelNonUniformityNormalized  wavelet-LHL_firstorder_Median  wavelet-LHL_glcm_InverseVariance  wavelet-LHL_glrlm_RunVariance  wavelet-LHL_glszm_SizeZoneNonUniformityNormalized  wavelet-LHH_glcm_Imc1  wavelet-HLL_glrlm_LongRunEmphasis  wavelet-HLL_glszm_SmallAreaEmphasis  wavelet-HLH_glcm_DifferenceAverage  wavelet-HLH_glcm_Imc1  wavelet-HLH_glcm_Imc2  wavelet-HHH_gldm_DependenceVariance  wavelet-HHH_glrlm_ShortRunLowGrayLevelEmphasis  wavelet-LLL_firstorder_10Percentile  wavelet-LLL_glcm_Imc1  wavelet-LLL_glrlm_ShortRunEmphasis  wavelet-LLL_glszm_LargeAreaLowGrayLevelEmphasis |  | original_shape_Flatness  original_shape_Maximum2DDiameterSlice  original_shape_Sphericity  wavelet-LLH_firstorder_Energy  wavelet-LLH_firstorder_Kurtosis  wavelet-LLH_gldm_DependenceVariance  wavelet-LLH_glrlm_RunLengthNonUniformityNormalized  wavelet-LLH_glrlm_RunVariance  wavelet-LLH_glszm_GrayLevelNonUniformity  wavelet-LLH_glszm_LargeAreaHighGrayLevelEmphasis  wavelet-LLH_ngtdm_Contrast  wavelet-LHL_glcm_MaximumProbability  wavelet-LHL_glrlm_LongRunEmphasis  wavelet-LHL_glszm_SizeZoneNonUniformityNormalized  wavelet-LHH_firstorder_Mean  wavelet-LHH_glcm_ClusterShade  wavelet-HLL_glszm_SizeZoneNonUniformityNormalized  wavelet-HLH_firstorder_Median  wavelet-HLH_glcm_DifferenceEntropy  wavelet-HLH_glcm_Imc1  wavelet-HLH_glcm_Imc2  wavelet-HHH_firstorder_Median  wavelet-HHH_gldm_DependenceVariance  wavelet-LLL_glcm_Imc1 |
| MI |  | original_shape_Sphericity  original_glcm_DifferenceEntropy  wavelet-LLH_firstorder_Mean  wavelet-LLH_firstorder_Uniformity  wavelet-LLH_glcm_DifferenceVariance  wavelet-LLH_glcm_JointEnergy  wavelet-LLH_glcm_MaximumProbability  wavelet-LLH_gldm_DependenceEntropy  wavelet-LLH_gldm_GrayLevelVariance  wavelet-LLH_gldm_LargeDependenceEmphasis  wavelet-LLH_gldm_LargeDependenceLowGrayLevelEmphasis  wavelet-LLH_glrlm_LongRunEmphasis  wavelet-LLH_glrlm_LongRunLowGrayLevelEmphasis  wavelet-LLH_glrlm_RunLengthNonUniformityNormalized  wavelet-LLH_glrlm_RunPercentage  wavelet-LLH_glrlm_ShortRunEmphasis  wavelet-LHL_glrlm_RunEntropy  wavelet-LHH_glcm_ClusterShade  wavelet-HLL_glszm_SmallAreaEmphasis  wavelet-HLH_glcm_Autocorrelation  wavelet-HLH_glcm_JointAverage  wavelet-HLH_glcm_SumAverage  wavelet-HLH_gldm_HighGrayLevelEmphasis  wavelet-HLH_gldm_LargeDependenceHighGrayLevelEmphasis  wavelet-HLH_gldm_LowGrayLevelEmphasis  wavelet-HLH_glrlm_ShortRunHighGrayLevelEmphasis  wavelet-HHH_gldm_SmallDependenceEmphasis |  | wavelet-LLH_firstorder_Entropy  wavelet-LLH_firstorder_Median  wavelet-LLH_gldm_LargeDependenceLowGrayLevelEmphasis  wavelet-LLH_gldm_LowGrayLevelEmphasis  wavelet-LLH_glrlm_LongRunLowGrayLevelEmphasis  wavelet-LLH_glrlm_RunPercentage  wavelet-LLH_glszm_GrayLevelNonUniformity  wavelet-LLH_glszm_GrayLevelNonUniformityNormalized  wavelet-LHL_gldm_LowGrayLevelEmphasis  wavelet-LHH_firstorder_Mean  wavelet-LHH_glcm_ClusterShade  wavelet-HLL_glcm_ClusterShade  wavelet-HLH_glcm_ClusterShade  wavelet-HLH_glszm_GrayLevelNonUniformity |
| RFE |  | original_shape_Sphericity  original_firstorder_Minimum  wavelet-LLH_glcm_JointEnergy  wavelet-LLH_glcm_MaximumProbability  wavelet-LLH_gldm_DependenceEntropy  wavelet-LLH_glrlm_LongRunLowGrayLevelEmphasis  wavelet-LHL_glrlm_RunEntropy  wavelet-LHH_firstorder_Mean  wavelet-HLL_glszm_SizeZoneNonUniformityNormalized  wavelet-HLL_glszm_SmallAreaEmphasis  wavelet-HLH_glcm_DifferenceAverage  wavelet-HLH_glcm_DifferenceEntropy  wavelet-HLH_glcm_DifferenceVariance  wavelet-HLH_glcm_JointAverage  wavelet-HLH_glcm_SumAverage  wavelet-HLH_gldm_LargeDependenceHighGrayLevelEmphasis  wavelet-HLH_gldm_SmallDependenceHighGrayLevelEmphasis  wavelet-HLH_glrlm_ShortRunHighGrayLevelEmphasis  wavelet-HLH_glrlm_ShortRunLowGrayLevelEmphasis  wavelet-HHH_gldm_DependenceEntropy  wavelet-LLL_firstorder_10Percentile  wavelet-LLL_glcm_DifferenceVariance |  | original_shape_Sphericity  wavelet-LLH_firstorder_Uniformity  wavelet-LLH_glcm_MaximumProbability  wavelet-LLH_gldm_LargeDependenceEmphasis  wavelet-LLH_glrlm_RunLengthNonUniformityNormalized  wavelet-LLH_glrlm_ShortRunEmphasis  wavelet-LLH_glszm_GrayLevelNonUniformity  wavelet-LHL_gldm_DependenceNonUniformityNormalized  wavelet-LHH_firstorder_Median  wavelet-LHH_glcm_ClusterShade  wavelet-HLL_glcm_ClusterShade  wavelet-HLL_glszm_SmallAreaEmphasis  wavelet-HLH_firstorder_Median  wavelet-HLH_glcm_ClusterShade  wavelet-HLH_glcm_DifferenceEntropy  wavelet-HLH_glcm_Imc2  wavelet-HLH_gldm_LowGrayLevelEmphasis  wavelet-HLH_glrlm_LowGrayLevelRunEmphasis  wavelet-HHH_gldm_DependenceEntropy  wavelet-HHH_gldm_DependenceVariance  wavelet-HHH_gldm_SmallDependenceLowGrayLevelEmphasis  wavelet-LLL_glcm_MCC |
|  |  |  |  |  |
|  |  | Plain scan |  | Three phases |
| Lasso |  | original_shape_Flatness  original_shape_Sphericity  original_shape_SurfaceVolumeRatio  original_firstorder_Skewness  original_firstorder_Uniformity  original_glszm_GrayLevelNonUniformity  wavelet-LLH_firstorder_90Percentile  wavelet-LLH_glcm_DifferenceEntropy  wavelet-LLH_glrlm_RunEntropy  wavelet-LLH_glrlm_RunVariance  wavelet-LLH_glrlm_ShortRunEmphasis  wavelet-LLH_glrlm_ShortRunLowGrayLevelEmphasis  wavelet-LLH_glszm_GrayLevelNonUniformity  wavelet-LLH_glszm_GrayLevelVariance  wavelet-LLH_glszm_LargeAreaLowGrayLevelEmphasis  wavelet-LLH_ngtdm_Busyness  wavelet-LHL_firstorder_Mean  wavelet-LHL_firstorder_Median  wavelet-LHL_glcm_MCC  wavelet-LHL_gldm_LargeDependenceLowGrayLevelEmphasis  wavelet-LHL_glrlm_LongRunEmphasis  wavelet-LHL_glrlm_RunLengthNonUniformityNormalized  wavelet-LHL_glrlm_RunVariance  wavelet-LHL_glszm_LowGrayLevelZoneEmphasis  wavelet-LHL_glszm_SmallAreaEmphasis  wavelet-LHL_glszm_SmallAreaHighGrayLevelEmphasis  wavelet-LHL_glszm_ZonePercentage  wavelet-LHL_glszm_ZoneVariance  wavelet-LHH_firstorder_Mean  wavelet-LHH_firstorder_Uniformity  wavelet-LHH_glcm_ClusterShade  wavelet-LHH_glcm_SumSquares  wavelet-LHH_gldm_LowGrayLevelEmphasis  wavelet-LHH_glszm_HighGrayLevelZoneEmphasis  wavelet-HLL_firstorder_Mean  wavelet-HLL_firstorder_Median  wavelet-HLL_glcm_ClusterShade  wavelet-HLL_gldm_LargeDependenceEmphasis  wavelet-HLL_glrlm_LongRunEmphasis  wavelet-HLL_glszm_LargeAreaLowGrayLevelEmphasis  wavelet-HLH_firstorder_10Percentile  wavelet-HLH_glcm_DifferenceEntropy  wavelet-HLH_glcm_Imc1  wavelet-HLH_glcm_Imc2  wavelet-HHL_firstorder_Mean  wavelet-HHL_gldm_DependenceNonUniformityNormalized  wavelet-HHH_gldm_DependenceVariance  wavelet-HHH_glrlm_RunLengthNonUniformityNormalized  wavelet-HHH_glrlm_RunVariance  wavelet-LLL_firstorder_Minimum  wavelet-LLL_glcm_ClusterShade  wavelet-LLL_glcm_Imc1  wavelet-LLL_glcm_InverseVariance  wavelet-LLL_glrlm_RunLengthNonUniformity  wavelet-LLL_glrlm_ShortRunEmphasis  wavelet-LLL_glszm_GrayLevelNonUniformity  wavelet-LLL_ngtdm_Contrast |  | A_original_shape_Elongation  A_original_shape_Sphericity  A_wavelet-LLH_firstorder_Kurtosis  A_wavelet-LLH_glrlm_LongRunLowGrayLevelEmphasis  A_wavelet-LLH_glszm_GrayLevelNonUniformityNormalized  A_wavelet-LHL_glcm_InverseVariance  A_wavelet-LHL_gldm_DependenceVariance  A_wavelet-LHL_glszm_SizeZoneNonUniformityNormalized  A_wavelet-LHH_glcm_Imc1  A_wavelet-LHH_glrlm_RunVariance  A_wavelet-HLL_firstorder_Mean  A_wavelet-HLL_glszm_SmallAreaEmphasis  A_wavelet-HHH_gldm_DependenceVariance  A_wavelet-LLL_firstorder_Minimum  A_wavelet-LLL_glszm_LargeAreaLowGrayLevelEmphasis  V_original_shape_Maximum2DDiameterSlice  V_original_glszm_GrayLevelNonUniformityNormalized  V_wavelet-LLH_firstorder_Energy  V_wavelet-LLH_gldm_DependenceVariance  V_wavelet-LLH_glrlm_RunLengthNonUniformity  V_wavelet-LLH_glrlm_RunVariance  V_wavelet-LLH_glszm_GrayLevelNonUniformity  V_wavelet-LLH_glszm_GrayLevelNonUniformityNormalized  V_wavelet-LLH_ngtdm_Contrast  V_wavelet-LHL_glszm_SizeZoneNonUniformityNormalized  V_wavelet-LHH_glcm_ClusterProminence  V_wavelet-LHH_glcm_SumEntropy  V_wavelet-HLL_glszm_SizeZoneNonUniformity  V_wavelet-HLH_firstorder_Median  V_wavelet-HLH_glcm_Imc1  V_wavelet-HLH_glcm_Imc2  V_wavelet-HHH_firstorder_Median  V_wavelet-HHH_gldm_DependenceVariance  V_wavelet-LLL_firstorder_Skewness  V_wavelet-LLL_glcm_Imc1  V_wavelet-LLL_glcm_MCC  P_original_shape_Flatness  P_original_firstorder_Minimum  P_wavelet-LLH_glrlm_RunEntropy  P_wavelet-LLH_glrlm_ShortRunLowGrayLevelEmphasis  P_wavelet-LLH_glszm_LargeAreaLowGrayLevelEmphasis  P_wavelet-LLH_glszm_LowGrayLevelZoneEmphasis  P_wavelet-LHL_firstorder_Median  P_wavelet-LHL_glcm_MCC  P_wavelet-LHL_glrlm_RunLengthNonUniformityNormalized  P_wavelet-LHL_glrlm_RunVariance  P_wavelet-LHL_glszm_LargeAreaLowGrayLevelEmphasis  P_wavelet-LHL_glszm_SmallAreaEmphasis  P_wavelet-LHH_glcm_ClusterShade  P_wavelet-LHH_glrlm_LongRunHighGrayLevelEmphasis  P_wavelet-HLL_firstorder_Mean  P_wavelet-HLL_glcm_ClusterShade  P_wavelet-HLL_glszm_LargeAreaLowGrayLevelEmphasis  P_wavelet-HLH_glcm_DifferenceEntropy  P_wavelet-HLH_glcm_Imc2  P_wavelet-HHL_firstorder_Mean  P_wavelet-HHL_glrlm_LongRunEmphasis  P_wavelet-HHH_gldm_DependenceVariance  P_wavelet-HHH_glrlm_RunLengthNonUniformityNormalized  P_wavelet-LLL_glcm_Idn  P_wavelet-LLL_glcm_Imc1  P_wavelet-LLL_ngtdm_Contrast |
| MI |  | original_glcm_ClusterShade  wavelet-LLH_glcm_DifferenceAverage  wavelet-LLH_glcm_Id  wavelet-LLH_glcm_Idm  wavelet-LLH_glcm_InverseVariance  wavelet-LLH_glcm_JointEnergy  wavelet-LLH_gldm_DependenceEntropy  wavelet-LLH_gldm_LargeDependenceEmphasis  wavelet-LLH_gldm_LargeDependenceLowGrayLevelEmphasis  wavelet-LLH_gldm_LowGrayLevelEmphasis  wavelet-LLH_gldm_SmallDependenceLowGrayLevelEmphasis  wavelet-LLH_glrlm_LongRunEmphasis  wavelet-LLH_glrlm_LongRunLowGrayLevelEmphasis  wavelet-LLH_glrlm_RunPercentage  wavelet-LLH_glrlm_RunVariance  wavelet-LLH_glrlm_ShortRunLowGrayLevelEmphasis  wavelet-LHL_firstorder_Median  wavelet-LHL_glcm_JointAverage  wavelet-LHL_glcm_SumAverage  wavelet-LHL_gldm_DependenceNonUniformityNormalized  wavelet-LHL_gldm_LargeDependenceLowGrayLevelEmphasis  wavelet-LHH_glcm_Autocorrelation  wavelet-LHH_glcm_ClusterShade  wavelet-LHH_glcm_JointAverage  wavelet-LHH_glcm_SumAverage  wavelet-LHH_gldm_HighGrayLevelEmphasis  wavelet-LHH_gldm_LargeDependenceHighGrayLevelEmphasis  wavelet-LHH_gldm_LowGrayLevelEmphasis  wavelet-LHH_glrlm_ShortRunHighGrayLevelEmphasis  wavelet-LHH_glszm_HighGrayLevelZoneEmphasis  wavelet-HLL_firstorder_Median  wavelet-HLH_gldm_LowGrayLevelEmphasis  wavelet-HHL_glrlm_LongRunEmphasis  wavelet-LLL_firstorder_Maximum  wavelet-LLL_firstorder_RootMeanSquared  wavelet-LLL_glcm_ClusterShade |  | A_original_shape_Sphericity  A_wavelet-LLH_firstorder_Entropy  A_wavelet-LLH_glcm_Autocorrelation  A_wavelet-LLH_glcm_JointEnergy  A_wavelet-LLH_glcm_MaximumProbability  A_wavelet-LLH_glcm_SumEntropy  A_wavelet-LLH_gldm_GrayLevelVariance  A_wavelet-LLH_gldm_LargeDependenceEmphasis  A_wavelet-LLH_gldm_LargeDependenceLowGrayLevelEmphasis  A_wavelet-LLH_gldm_LowGrayLevelEmphasis  A_wavelet-LLH_glrlm_LongRunLowGrayLevelEmphasis  A_wavelet-LLH_glrlm_RunLengthNonUniformityNormalized  A_wavelet-LLH_glrlm_RunPercentage  A_wavelet-HLH_glcm_Autocorrelation  A_wavelet-HLH_glcm_JointAverage  A_wavelet-HLH_glcm_SumAverage  A_wavelet-HLH_gldm_LargeDependenceHighGrayLevelEmphasis  A_wavelet-HHL_glrlm_RunPercentage  V_wavelet-LLH_gldm_LargeDependenceLowGrayLevelEmphasis  V_wavelet-LLH_glrlm_LongRunLowGrayLevelEmphasis  P_wavelet-LLH_glcm_JointEnergy  P_wavelet-LLH_gldm_DependenceEntropy  P_wavelet-LLH_gldm_LargeDependenceEmphasis  P_wavelet-LLH_gldm_LargeDependenceLowGrayLevelEmphasis  P_wavelet-LLH_gldm_SmallDependenceLowGrayLevelEmphasis  P_wavelet-LLH_glrlm_LongRunEmphasis  P_wavelet-LLH_glrlm_RunPercentage  P_wavelet-LLH_glrlm_ShortRunLowGrayLevelEmphasis  P_wavelet-LHL_firstorder_Median  P_wavelet-LHL_glcm_JointAverage  P_wavelet-LHL_glcm_SumAverage  P_wavelet-LHL_gldm_LargeDependenceLowGrayLevelEmphasis  P_wavelet-LHH_glcm_Autocorrelation  P_wavelet-LHH_glcm_JointAverage  P_wavelet-LHH_glcm_SumAverage  P_wavelet-LHH_glrlm_ShortRunHighGrayLevelEmphasis  P_wavelet-LHH_glszm_HighGrayLevelZoneEmphasis |
| RFE |  | original_shape_Sphericity  original_firstorder_Minimum  original_firstorder_Skewness  wavelet-LLH_glcm_JointEnergy  wavelet-LLH_glcm_MaximumProbability  wavelet-LLH_gldm_LargeDependenceEmphasis  wavelet-LLH_glrlm_LongRunEmphasis  wavelet-LLH_glrlm_ShortRunLowGrayLevelEmphasis  wavelet-LLH_glszm_LargeAreaLowGrayLevelEmphasis  wavelet-LHL_firstorder_Median  wavelet-LHL_glszm_SmallAreaEmphasis  wavelet-LHH_glcm_ClusterShade  wavelet-LHH_glcm_JointAverage  wavelet-LHH_glcm_SumAverage  wavelet-LHH_gldm_LargeDependenceEmphasis  wavelet-LHH_glrlm_ShortRunHighGrayLevelEmphasis  wavelet-HLH_firstorder_10Percentile  wavelet-HLH_glcm_Imc1  wavelet-HHL_glrlm_LongRunEmphasis  wavelet-LLL_firstorder_Minimum  wavelet-LLL_glcm_ClusterShade  wavelet-LLL_glrlm_RunLengthNonUniformity |  | A_original_shape_Sphericity  A_wavelet-LLH_firstorder_Entropy  A_wavelet-LLH_firstorder_Maximum  A_wavelet-LLH_glrlm_LongRunEmphasis  A_wavelet-LLH_glrlm_LongRunLowGrayLevelEmphasis  A_wavelet-LLH_glrlm_RunVariance  A_wavelet-LLH_glszm_GrayLevelNonUniformity  A_wavelet-HLH_glcm_JointAverage  A_wavelet-HLH_glcm_SumAverage  A_wavelet-HLH_glrlm_LongRunHighGrayLevelEmphasis  A_wavelet-HHH_gldm_SmallDependenceEmphasis  A_wavelet-HHH_glrlm_ShortRunLowGrayLevelEmphasis  V_wavelet-HLH_firstorder_Median  V_wavelet-HLH_glcm_ClusterShade  V_wavelet-HLH_glrlm_ShortRunLowGrayLevelEmphasis  V_wavelet-LLL_glcm_MCC  P_original_shape_Sphericity  P_original_firstorder_Minimum  P_wavelet-LLH_glrlm_RunPercentage  P_wavelet-LHH_firstorder_Mean  P_wavelet-LHH_glcm_Autocorrelation  P_wavelet-LHH_glcm_ClusterShade  P_wavelet-LHH_glcm_JointAverage  P_wavelet-LHH_gldm_LargeDependenceHighGrayLevelEmphasis  P_wavelet-HHL_gldm_DependenceVariance |

Note: Lasso, least absolute shrinkage and selection operator; MI, mutual information; RFE, recursive feature elimination
